# A novel RRGW derived peptide is a promising inhibitor of BoNT/A

**DOI:** 10.1080/14756366.2023.2203878

**Published:** 2023-04-27

**Authors:** Wantong Ma, Lulu Wang, Xiangmin Tan, Xin Wang, Chunyan Yang, Yu Wang, Ziye Liu, Bo Liu, Hai Zhu, Dejuan Zhi, Dongsheng Wang

**Affiliations:** a School of Pharmacy, Lanzhou University, Lanzhou, P.R. China; b Frontiers Science Center for Rare Isotopes, Lanzhou University, Lanzhou, P.R. China; c State Key Laboratory for Animal Disease Control and Prevention, College of Veterinary Medicine, Lanzhou University, Lanzhou, P.R. China

**Keywords:** Botulinum neurotoxins A, Inhibitors, Derived peptide, Dynamics simulations

## Abstract

Clostridium botulinum neurotoxin type A (BoNT/A) is one of the most potent biotoxins ever known. Its entry into neurons could block vesicle exocytosis to abolish the release of neurotransmitters from nerve terminals, thus leading to muscle paralysis. Although there are so many peptides, antibodies and chemical compounds claimed to have anti-toxin activity, no drug is available in the clinical application except equine antitoxin serum. In the present work, a short peptide inhibitor RRGW of BoNT/A was firstly identified by computer-aided ligand-receptor binding simulation, then an RRGW derived peptide was rational designed based on the fragment of SNAP-25 (141–206 aa). Proteolytic assay showed that the anti-toxin activity of the RRGW derived peptide was much higher than that of RRGW. Digit abduction score assay demonstrated that the derived peptide delayed BoNT/A-induced muscle paralysis at a lower concentration by 20-fold than RRGW. The results supported that RRGW derived peptide can be a potential BoNT/A inhibitor candidate for further treating botulism.

## GRAPHICAL ABSTRACT

**Figure:**
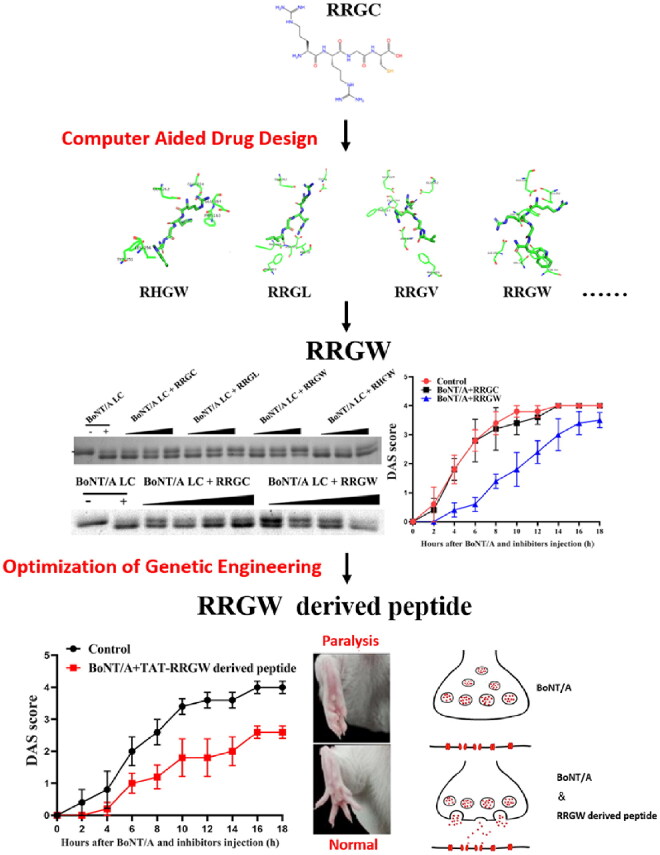

## Introduction

Botulinum neurotoxins (BoNTs) produced by the bacteria *Clostridium botulinum*, are the most toxic biotoxins that can cause flaccid paralysis leading to death^1^. According to their immunological characteristics, there are seven serotypes of botulinum neurotoxins, designated from BoNT/A to BoNT/G^2^. The toxins can bind to the specific neuronal receptors in the neuromuscular junctions (NMJs), where they cleave the soluble N-ethylmaleimide-sensitive factor attachment receptor (SNARE) proteins to block acetylcholine release and consequently cause flaccid paralysis^3^. BoNTs are generally composed of a heavy chain (HC, 100 KDa) and a light chain (LC, 50 KDa) covalently linked by a disulphide bond. The HC is responsible for binding to the receptor on the surface of the neuronal cells, and then assisting the LC to translocate into the cytosol, where the SNARE proteins serve as the relative substrates of the LCs. The synaptosome-associated 25 KDa protein (SNAP-25) can be cleaved by BoNT/A and E^4^, VAMP2 can be cleaved by BoNT/B, D and G, and both SNAP-25 and syntaxin can be cleaved by BoNT/C.

BoNT/A is the most common pathogenic BoNT serotype in human and is one of the most poisonous toxins that ever known. It causes muscle paralysis and lasts for months, even leads to death. BoNT/A could be potentially used as bioweapon, so it has been classified as Category A biowarfare agent by the Centres for Disease Control and Prevention (CDC) in the United States^5^. Owing to its special potent pharmacological activity and long-lasting effects, BoNT/A has been frequently used in clinical practices to treat different diseases associated with dystonia and to reduce facial wrinkles in cosmetic applications. In recent years, the rapid expansion of BoNT/A in use caused more and more intoxication cases and adverse effects. 217 serious and 189 nonserious cases followed therapeutic use of BoNT/A, and 36 serious and 995 nonserious cases followed cosmetic use were reported to the FDA in only a year of 2005^6^. Hence, the effective antidotes became urgent need to break through the limitation of BoNT/A.

Currently, there are only some antitoxins approved to neutralise BoNT/A against botulism. However, once the toxins translocate into the cytosol of neuronal cells, the antibody antitoxins cannot block the flaccid paralysis in the patient due to poor cellular entry ability of antibodies, so this intervention is only partially effective within 12–24 h post-exposure to the toxin^7^. Some small molecules, such as quinolinol compounds have been reported to mitigate the toxicity of BoNT/A in neuronal cells and reduce proteolytic activity in molecular level^8^. Several other molecules have been investigated to act as BoNT/A inhibitors, such as BoNT/A light chain α and β-exosite binders, translocation blockers, and Kv channel blockers^9^. Although these molecules could directly or competitively inhibit BoNT/A light chain proteolytic activity, there are still no FDA-approved drugs to treat BoNT-induced paralysis after BoNT/A intoxication^10^. Therefore, it is imperative to develop specific inhibitors against BoNT/A intoxication.

Fortunately, small peptide inhibitors, such as CPI-I, RRGC and GRKKRRQRRRPPQC offer a new sight in developing specific inhibitors for BoNT/A intoxication therapy^11^. In this paper, a new RRGW derived peptide with better inhibitory performance on BoNT/A was reported, it acted as a kind of anti-toxin agent by binding to the active site of the internalised BoNT/A light chain and reduced the proteolytic activity of BoNT/A. On an organism level, it delayed BoNT/A-induced muscle paralysis, especially when co-treatment with BoNT/A.

## Methods and materials

### Animals, toxins, and other materials

Mice were obtained from GLP laboratory in Lanzhou Veterinary Research Institute, Chinese Academy of Agricultural Sciences. BoNT/A were provided by Lanzhou Institute of Biological Products Co., Ltd. Short peptide inhibitors and the synthetic substrate were synthesised by Dan Gang Peptides Co., Ltd. The peptides were routinely purified and analysed by HPLC and MS with purities more than 95%. The RRGW-derived peptide was genetically recombined as the fragment of SNAP-25 (141–206 aa) and fused with TAT at its N-terminal and His tags at its C-terminal. The molecular weight of the desired peptide is 11.19 kDa, and it was purified by Ni^2+^ affinity chromatography by using a binding buffer contained 50 mM PB pH 7.4, 300 mM NaCl, and 50 mM imidazole, and an elution buffer contained 50 mM PB pH 7.4, 300 mM NaCl, and 300 mM imidazole.

### Cell culture

SH-SY5Y and Neuro-2A (N2A) cell lines were cultured in DMED solution containing 10% FBS and 1% antibiotics. All the cultures were incubated at 37 °C and 5% CO_2_. All experiments were done using cells of passage number 4 to 20.

### Design and homology modelling of polypeptide inhibitor

The X-ray crystal structure of BoNT/A LC in complex with RRGC peptide (PDB ID:3C88) was downloaded from RCSB database. Then, a virtual pool of 59 peptides analogous to RRGC was designed by replacing the amino acids of RRGC with other amino acids to enhance the BoNT/A light chain binding affinity. Discovery Studio 2.5 software was used to complete the amino acid replacement to obtain the newly designed 3D conformations of basic tetrapeptide.

### Molecular docking

The binding affinity of the basic tetrapeptide to BoNT/A LC was predicted by molecular docking. The docking method was carried out by applying two docking methods of ZDock and AutoDock. The ZDock module was carried out by molecular docking with the Discovery Studio 2.5. ZDock is based on the rigid-body protein-protein docking program that uses the Fast Fourier Transform algorithm to enable an efficient global docking. To improve the accuracy of the docking, the Euler angle was set at 6° in this docking to obtain 54,000 bonding configurations. Semi-flexible docking was operated through Autodock 4 software. The Autodock tool initialises the ligand by adding gasteiger charges, merging non-polar hydrogen bonds, and setting rotatable bonds. The BoNT/A LC and tetrapeptide ligand file were saved in PDBQT format and used for docking. Autodock Tools were used to add polar hydrogen and Gasteiger charge to the entire BoNT/A LC. Set the docking centre to (20.787, 16.672, 58.408), the number of box grid points is X = Y = Z = 72, the grid spacing is 0.375 Å, and the number of collisions was set to 50 to improve the docking accuracy. The grid box was set to contain the entire BoNT/A LC region and the grid spacing is 0.375 Å. For docking, BoNT/A LC was held fixed and the ligand remained flexible. Finally, the best conformation from the molecular docking model with the lowest binding energy score was selected for the next analysis.

### Dynamics simulations

In AMBER 14 software, the t-leap module was designed to generate the topology files and coordinate files of the systems. All systems used the standard FF14SB force field to generate the composite force fields^12^. And all systems were wrapped in a cube box of TIP3P water model, with each amino acid in the complex at least 10 Å away from the edge of the water box. The corresponding amount of Cl^−^ was added into the whole system to ensure the electrical neutrality of the system. Energy minimisation, thermalised and equilibrated of the system is accomplished through the Sander program in AMBER14.

For energy minimisation, all the atoms were firstly constrained by 5.0 kcal·mol^−1^·Å^−2^ and executed 5000 steps. Then, the protein atoms were constrained by 3.0 kcal·mol^−1^·Å^−2^ to make the solvent molecules reach the proper position and executed 5000 steps. The last minimum was executed without any constraints.

In the minimisation process, the first 2500 steps of steepest descent were performed, and conjugate gradient method was used for the last 2500 steps. After energy minimisation, the system was gradually heated from 0 K to 310 K in a constant temperature and constant volume (NVT) system, which lasted for 100 ps. During the heating process, a limiting force was applied to all atoms in the complex, and the limiting force constant was set at 5 kcal mol^−1 ^Å^−2^. The entire system was run under a constant NPT system for a 1.5 ns equilibrium simulation. The first 1.0 ns was divided into 5 stages where the limiting force gradually decreased. Each stage ran for 200 ps, and the limiting force was 5.0 kcal·mol^−1^·Å^−2^, 4.0 kcal·mol^−1^·Å^−2^, 3.0 kcal·mol^−1^·Å^−2^, 2.0 kcal·mol^−1^·Å^−2^, 1.0 kcal·mol^−1^·Å^−2^, respectively. The last 500ps were carried out without any restraint. Finally, a 200 ns production of MD simulation of each system was performed at a temperature of 310 K and a pressure of 1 atm in the NPT ensemble without any restraint. In the simulation, the SHAKE algorithm was used to limit the bond length involving hydrogen and the Particle Mesh Ewald (PME) was used to handle the long-range Coulomb interactions, and set the non-bond cut off value to 10 Å to deal with non-bond interaction^13^. Periodic boundary conditions were applied to avoid edge effects. In AMBER14, the MM-GBSA method was used to calculate the binding free energy (ΔGbind) between the tetrapeptide inhibitors and BoNT/A LC. An average of 10,000 instantaneous structures was extracted from the last 50 ns stable MD trajectory at a time interval of 5 ps to calculate the binding free energy. Since the binding abilities of multiple different tetrapeptide inhibitors to the same protein were aimed to compared, the effect of entropy in the calculation process was ignored.

### MTT assay

About 1 × 10^4^ SH-SY5Y or N2A cells per well were seeded in 96-well plates for 12 h. Cells were treated with different concentrations of RRGC and RRGW for another 48 h. The cell culture medium was removed, 10 μL MTT (5 mg/mL) was added into each well of the 96-well plates, which were dissolved by 100 μL DMSO. Cell viability was calculated according to the formula = 1× (sample Abs)/(control Abs). Abs is the absorbance value at 490 nm.

### Proteolytic assays for the inhibitor activity with SNAP-25 in vitro

To evaluate whether the inhibitors can block the BoNT/A LC (1–448 aa) proteolytic activity, inhibitors and BoNT/A LC were co-incubated in the presence of SNAP-25^14^. Before each assay, 2 nM BoNT/A LC were added into master mix containing 2.5 μM SNAP-25 proteins, 0.25 mM ZnCl_2_ and 5 mM DTT, and incubated at 37 °C for 15 min. For rescue assays, inhibitors were added to the master mix at indicated different time point. All the reaction was stopped by adding loading buffer. The samples were analysed by SDS-PAGE on a 12% polyacrylamide gel, the gel was stained by Coomassie blue R250, and scanned by Tanon Digital Gel Analysis System.

### HPLC assays for the substrates and products

Recombinant BoNT/A LC (1–448 aa) was purified as described previously^15^. The SNAP-25 substrate peptide SNKTRIDEANQRATKML was custom synthesised and purified to more than 95% by Dan Gang Peptides Co., Ltd. Agilent 1260 HPLC system with Empower Pro-software were used to measure intact substrates and products (Diamonsil 5 μM C18, 250 × 4.6 mm). Solvent A was 0.1% trifluoroacetic acid in water and solvent B was 0.1% trifluoroacetic acid in 70% acetonitrile. The sample was injected after the column was equilibrated with 10% B on a flow rate of 1 ml/min at 25 °C. The column was held at 10% B for 2.5 min, and then run a linear gradient to 36% B over 23 min, finally performed 100% B for 6 min.

### Digit abduction score assay

KunMing (KM) mice were obtained from Lanzhou Veterinary Research Institute, Chinese Academy of Agricultural Sciences, and were randomly divided into indicated experiment groups as BoNT/A injection only, BoNT/A co-injection with RRGC, RRGW or RRGW derived peptide, repectively. Breifly, 0.75 U BoNT/A was co-injected with 10 μL inhibitor at indicated concentration or not into the gastrocnemius muscle of the left hinds. The degree of paralysis was scored in the next 18 h and statistically analysed.

### Statistical analysis

Statistical analysis was performed by the SPSS 19.0 software. Data were expressed as means ± SD. The differences were considered as statistically significant at *p* < 0.05.

## Results

### Design and homology modelling of short peptide inhibitor

Previously, RRGC tetrapeptide has been reported that can inhibit BoNT/A LC proteolytic activity by mimicking its substrate P1-P1'-P2'-P3' structure. So, a pool of basic polypeptide inhibitors was obtained by replacing the amino acid residue of RRGC. The P1 amino acid can be either R or C, because RRGC demonstrated higher activity than CRGC in *in vitro* experiments, whereas CRGC showed higher activity than RRGC in cell-based experiments. The P1' amino acid should have a positively charged group that can form a salt bridge with Asp370 of BoNT/A, so it can be substituted with K or M. According to existing literature, substituting the P2' amino acid with other amino acids leads to a decrease in inhibitor activity, so the P2' amino acid remains unchanged. The activity of RRGC decreases in the presence of DTT, so replacing the P3' C with a hydrophobic amino acid such as W/F/V/L/I/A/M/ORN (Ornithine)/CSE (Selenocysteine) can eliminate the effect of DTT and can therefore be substituted. Data was shown in Table 1.

**Table 1.** The pool of polypeptide inhibitors generated.

The amino acid sequences of modified basic polypeptides									
RRGC	RRGW	RRGF	RRGV	RRGL	RRGI	RRGA	RRGM	RRGORN	RRGCSE
CRGC	CRGW	CRGF	CRGV	CRGL	CRGI	CRGA	CRGM	CRGORN	CRGCSE
RKGC	RKGW	RKGF	RKGV	RKGL	RKGI	RKGA	RKGM	RKGORN	RKGCSE
RHGC	RHGW	RHGF	RHGV	RHGL	RHGI	RHGA	RHGM	RHGORN	RHGCSE
CKGC	CKGW	CKGF	CKGV	CKGL	CKGI	CKGA	CKGM	CKGORN	CKGCSE
CHGC	CHGW	CHGF	CHGV	CHGL	CHGI	CHGA	CHGM	CHGORN	CHGCSE

### RRGW showed a higher degree of energy contribution when acting as the ligand binds to BoNT/a LC

Through the ZDock module in Discovery Studio 2.5, BoNT/A LC and basic tetrapeptide inhibitors were molecularly docked, and 54,000 binding configurations were obtained for each system. The Z Rank Score indicated the degree of energy contribution to the system when the ligands bound to BoNT/A LC. The lower calculated value of ZRank Score certifies better the energy contributed to the system when the ligand bound to BoNT/A LC. The ZDock Score was calculated according to the shape matching of BoNT/A LC and ligand, and a higher score represented a more proper docking shape. The lower average value score in AutoDock was obtained, the better match between the ligands and BoNT/A LC occurred. In the process of screening docking results, the two scores to select peptide inhibitors with better docking results were comprehensively considered, and the screening results were shown in Table 2.

**Table 2.** Docking scores of polypeptide inhibitors with BoNT/A LC.

System	ZDock		System	AutoDock
	ZDock Score	ZRank Score (kcal/mol)		Average value
BoNT/A LC /RRGW	10.180	−97.749	BoNT/A LC /RKGW	−9.350
BoNT/A LC /RRGL	8.760	−91.384	BoNT/A LC /RHGW	−9.340
BoNT/A LC /RKGW	9.120	−91.298	BoNT/A LC /RRGW	−9.300
BoNT/A LC /RRGV	8.800	−86.496	BoNT/A LC /RKGF	−9.180
BoNT/A LC /RRGC	8.860	−86.158	BoNT/A LC /RRGF	−8.960
BoNT/A LC /RRGF	8.720	−85.266	BoNT/A LC /RRGL	−8.520
BoNT/A LC /RKGF	8.920	−83.692	BoNT/A LC /RRGA	−8.480
BoNT/A LC /RHGW	9.300	−82.498	BoNT/A LC /CRGF	−8.290
BoNT/A LC /CRGF	7.980	−80.674	BoNT/A LC /RRGC	−8.290
BoNT/A LC /RRGA	9.320	−80.391	BoNT/A LC /RRGV	−8.200

### The binding capability of RRGW was better than RRGC

**Figure 1.**
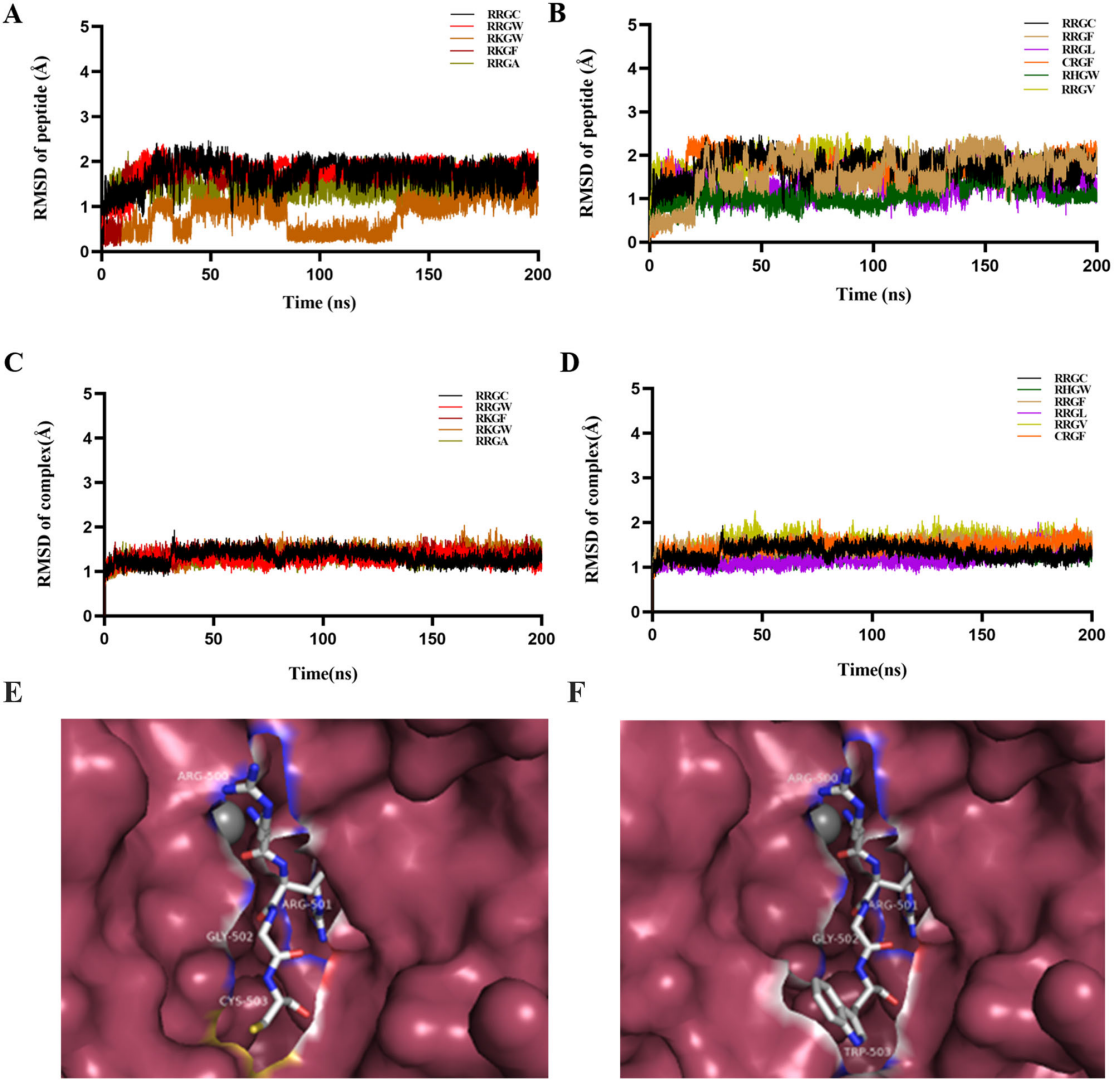
RMSD of the complex and polypeptide inhibitors. (A, B) The RMSD of the protein skeleton atoms of the polypeptide inhibitors; (C, D) The RMSD of the skeleton atoms of complex. (E) The receptor-ligand interaction of RRGC with the BoNT/A LC active site; (F) The receptor-ligand interaction of RRGW with the BoNT/A LC active site.

After a 200 ns molecular dynamic simulation was performed on the system, the root mean square deviation (RMSD) of the whole system was calculated to evaluate the stability of the entire simulation system. The result showed the RMSD values of the complexes and polypeptide inhibitors. Each system had a slight fluctuation in the initial stage of dynamic simulation, but in the last 50 ns, the RMSD value of each system tended to be stable (Figure 1).

The MM-GBSA method was used to calculate the binding free energy of each system to verify the affinity of the peptide inhibitor to BoNT/A LC. The average binding free energies of 10,000 instantaneous structures extracted in the last 50 ns of all research systems were calculated. ΔE_ele_ represented electrostatic force, ΔE_vdw_ represented van der Waals force, ΔG_GB_ represented the contribution of polar solvation free energy, ΔG_SA_ represented the contribution of non-polar solvation free energy, and ΔE_gas_ represented the contribution of gas change when forming a complex. ΔE_solv_ represented the contribution of the change of solvation free energy to binding free energy when forming complex, and ΔG_bind_ represented the free energy of binding. The result showed that the binding free energy of polypeptide inhibitor RRGW, RRGV, RRGA, RRGL with BoNT/A LC was lower than that of template RRGC with BoNT/A LC, so the binding capability of RRGW, RRGV, RRGA, RRGL with BoNT/A LC was larger than that of RRGC with BoNT/A LC (Table 3).

**Table 3.** The free energy of peptide inhibitors binding with BoNT/A LC and the energy contribution of different components.

Complexes	Contribution(kcal/mol)						
	ΔE_ele_	ΔE_vdw_	ΔG_GB_	ΔG_SA_	ΔE_gas_	ΔE_solv_	ΔG_bind_
BoNT/A LC/RRGW	−690.6635	−53.9707	697.7317	−8.364	−744.6342	689.3677	−55.2665
BoNT/A LC/RRGV	−601.0229	−53.3443	613.2911	−7.5206	−654.3672	605.7705	−48.5967
BoNT/A LC/RRGA	−731.9321	−37.2895	731.6535	−6.8674	−769.2216	724.7861	−44.4356
BoNT/A LC/RRGL	−626.2059	−40.8158	632.1347	−7.4922	−667.0217	624.6425	−42.3793
BoNT/A LC/RRGC	−701.3	−35.8663	701.5455	−6.7522	−737.1662	694.7934	−42.3729
BoNT/A LC/RRGF	−559.3412	−39.7051	568.5641	−6.9288	−599.0463	561.6354	−37.4109
BoNT/A LC/CRGF	−441.5345	−44.0944	456.5669	−7.3876	−485.629	449.1792	−36.4497
BoNT/A LC/RKGF	−675.2327	−36.5356	689.8373	−6.1246	−711.7683	683.7128	−28.0555
BoNT/A LC/RKGW	−593.0084	−48.1467	624.1964	−7.6093	−641.1552	616.5871	−24.5681
BoNT/A LC/RHGW	−480.2001	−40.7659	503.2493	−6.4276	−520.9661	496.8217	−24.1443

### RRGW peptide inhibitor showed better proteolysis inhibition effect on BoNT/A LC than RRGC in vitro

To investigate the cytotoxicity of RRGW and RRGC tetrapeptides, the MTT assays were used to determine the effects of RRGW and RRGC on SH-SY5Y and N2A cells. Results showed that both peptides had no cytotoxicity in either SH-SY5Y or N2A cells (Figure S1).

**Figure 2.**
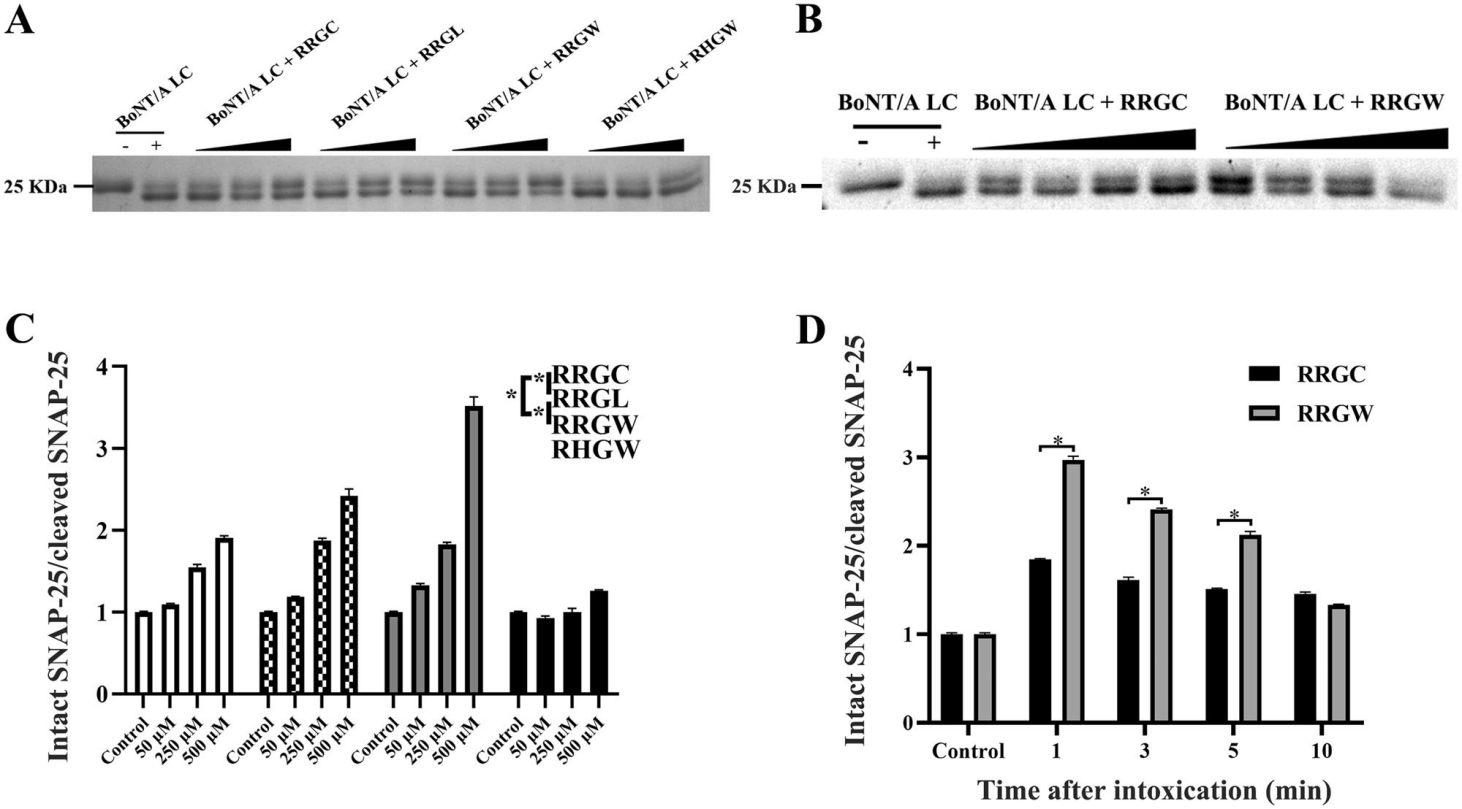
RRGW peptide showed higher inhibitory effect on BoNT/A LC proteolytic activities *in vitro*. SDS-PAGE results of intact and cleaved SNAP-25 in master mix after reaction with BoNT/A LC in the presence of inhibitors. The control refers to the group without inhibitor. (A) The protection effect of inhibitors after co-treated with BoNT/A at different concentrations; (B) The rescue effect of inhibitors in 1, 3, 5 and 10 min after exposure to BoNT/A LC; (C, D) Semi-quantification of intact SNAP-25 and cleaved SNAP-25 in A and B, respectively. The concentrations of inhibitors were 50 μM, 250 μM and 500 μM in A and C; and 500 μM RRGC and RRGW were used in B and D. The data are the average of three replicates, and * indicated there is significant difference between the treatment group and the control at *p* < 0.05.

BoNT/A LC and SNAP-25 were expressed in *E. coli* and purified using Ni^2+^ affinity chromatography as described previously (Figure S2)^15^. To evaluate the results of computer-aided molecular design, proteolytic activities of purified BoNT/A LC in the presence of peptide inhibitors or not were detected by substrate SNAP-25 cleavage as readout. The result showed that the ratio of intact SNAP-25 versus cleaved SNAP-25 protein increased in an inhibitor concentration-dependent manner. Consistent with the simulation result, RRGW showed a better inhibitory effect on BoNT/A proteolytic activity than RRGC, RRGL and RHGW at the same concentration (Figure 2(A,B)). Therefore, RRGW was selected for further comparison with RRGC in subsequent experiments. To test the rescue ability of RRGC and RRGW *in vitro*, inhibitors were added into SNAP-25 containing master mix after BoNT/A LC treatment for 1, 3, 5 and 10 min. Consequently, both RRGC and RRGW partially rescued SNAP-25 protein from BoNT/A LC cleavage at least in 5 min post intoxication. In addition, RRGW revealed a more significant inhibitory effect on BoNT/A to rescue SNAP-25 protein than RRGC (Figure 2(C,D)). These results showed that RRGW was superior to RRGC in inhibiting BoNT/A proteolytic activity on a molecular level.

### Injection of RRGW as inhibitor delayed BoNT/A-induced leg muscle paralysis

**Figure 3.**
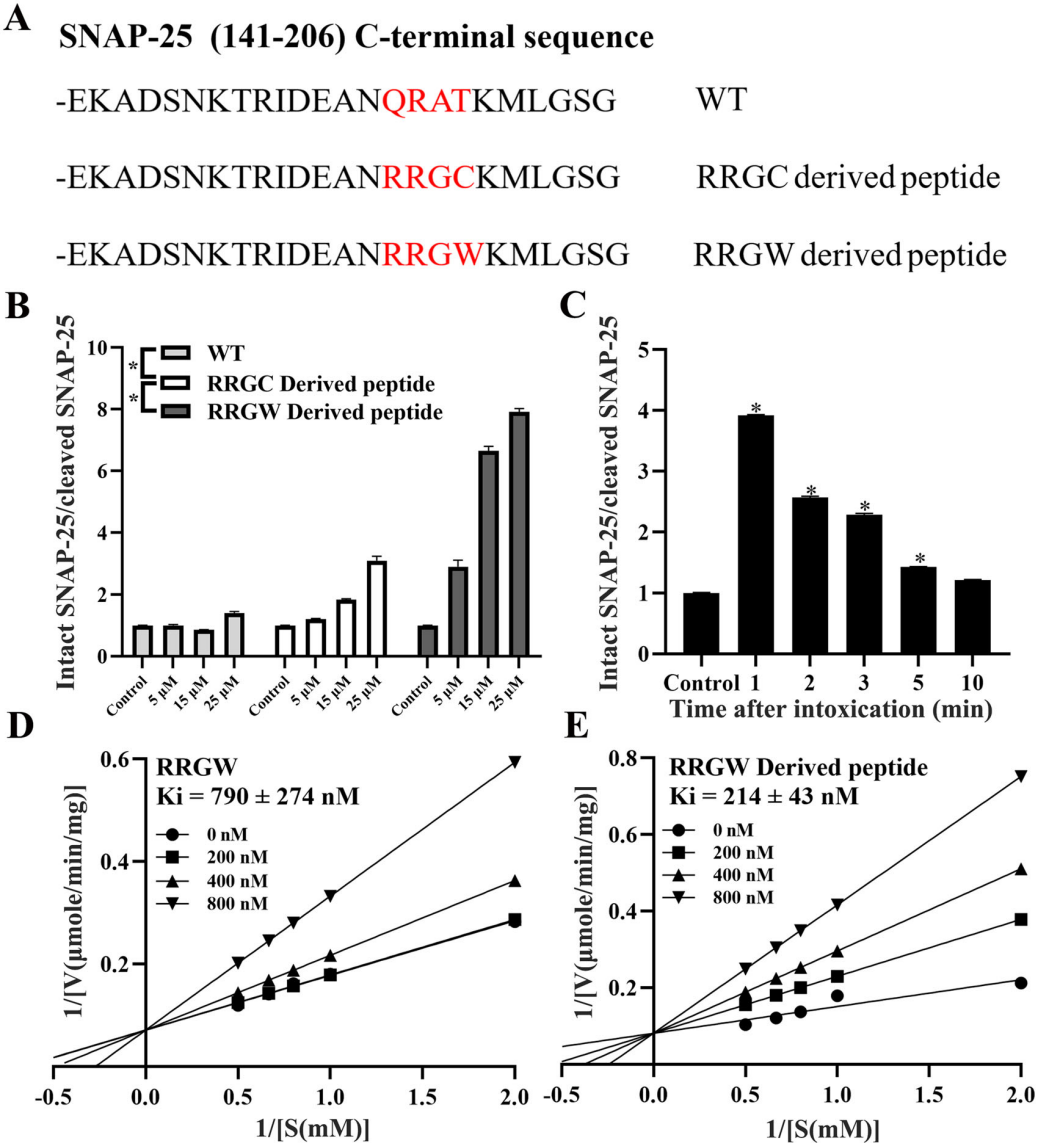
RRGW derived peptide showed higher inhibitory effect on BoNT/A LC proteolytic activities *in vitro*. (A) C-terminal sequence of different derived peptide; (B) The protection effect of inhibitors after co-treated with BoNT/A at different concentrations; (C) The rescue effect of RRGW derived peptide in 1, 2, 3, 5 and 10 min after exposure to BoNT/A LC; Double-reciprocal plots of velocity versus substrate concentration at several fixed concentration of RRGW (D) and RRGW derived peptide (E). The data are the average of three replicates, and * indicated there is significant difference between the treatment group and the control at *p* < 0.05.

To further assess activity of RRGW and RRGC *in vivo*, mice Digit Abduction Score (DAS) assay was used to test whether RRGW inhibited the paralysis effect of the local BoNT/A injection^16^. Several doses of BoNT/A were first injected intramuscularly into the hind legs of KM mice and the DAS scores were recorded. The results showed that 0.75 U toxins consistently caused the limb paralysis with little variation among the individuals at 14 to 18 h (Figure S3). So, 0.75 U was chosen for further experiment, muscle paralysis with DAS of 3–4 was induced repeatedly at 14 h after toxin injection in mice. When co-injected with BoNT/A, RRGW significantly delayed toxin-induced leg muscle paralysis (Figure 3(A,B)), RRGC did not delay leg muscle paralysis at all (Figure 3(A)). When injected after BoNT/A exposure, both RRGC and RRGW could not rescue the toxin-induced paralysis (Figure S4).

### RRGW-derived peptide showed better protease inhibition activity in vitro

Compared to QRAT of SNAP-25 located at the protease cut site by BoNT/A, docking result suggested that RRGC and RRGW may display stronger binding stability than QRAT. So, QRAT in the fragment of SNAP-25 (141–206 aa) peptide was replaced with RRGW or RRGC, and mutant RRGW derived peptide and RRGC derived peptide were obtained. The C-terminal sequences of the wild type polypeptide (WT) and the two mutant peptides are shown in Figure 3(A). In order to test the inhibitory effects of the derived peptides on BoNT/A LC, SNAP-25 cleaved assay was carried out. The results demonstrated that RRGW derived peptide significantly inhibited the activity of BoNT/A LC at a concentration by 25-fold lower than RRGW. In comparison, RRGC derived peptide has very limited improved inhibitory effect than RRGW (Figure 3(B)). Therefore, RRGW derived peptide was chosen for the following assays.

The rescue ability of RRGW derived peptide was investigated after pre-incubation of SNAP-25 with BoNT/A LC for 1, 2, 3, 5 and 10 min. The results showed that RRGW derived peptide only at a concentration of 25 μM significantly blocked BoNT/A LC cleaving its substrate (Figure 3(C)). HPLC assay results showed that the Ki of RRGW derived peptide was 214 ± 43 nM, whereas the Ki of RRGW was 790 ± 274 nM (Figure 3(D,E)).

### Injection of RRGW derived peptide as more effective inhibitor delayed BoNT/A-induced leg muscle paralysis

**Figure 4.**
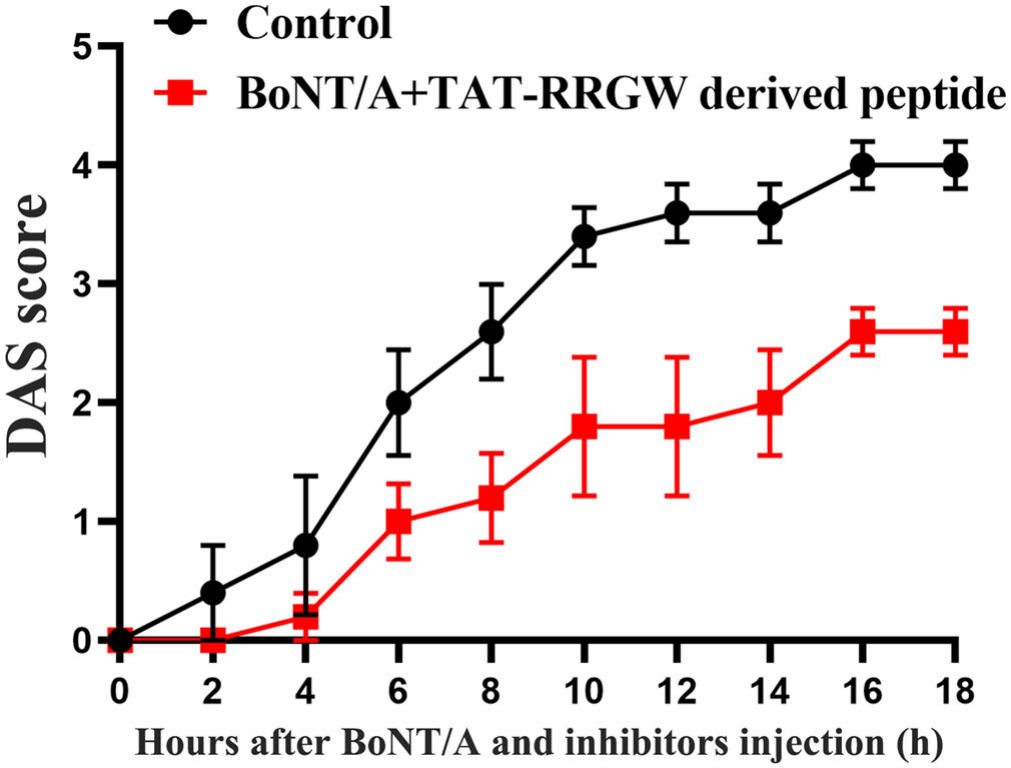
Co-injection of RRGW derived peptide with BoNT/A delayed BoNT/A-induced leg muscle paralysis. DAS score of the leg muscle paralysis progression within 18 h after injection with 0.75 U BoNT/A or co-injection with 0.75 U BoNT/A and 10 μL inhibitors. 25 μM RRGW derived peptide was used. There were five mice in each group, and * indicated there was significant difference between the treatment group and the control at *p* < 0.05.

DAS assay was further used to test the inhibitory effect of RRGW derived peptide on BoNT/A in mice. The result suggested that RRGW derived peptide displayed a similar effect to RRGW (Figure 4). It is deserved to note that RRGW derived peptide dosage used was lower than RRGW by 20-fold.

## Discussion

BoNT/A is widely used in medical and cosmetic practice, after all, it has been the most potent clostridial neurotoxins known and dangerous potential bioterrorism agents, there was insufficient medication to treat toxin poisoning^16^. There is only antitoxin is available to treat botulism so far. Small molecule inhibitors targeting the Zn^2+^ metalloprotease active site of toxin light chains have been successively discovered, including L-arginine oxo-hydroxy acid hydrochloride, Inh-1, Inh-2^8^^,^^17^, and 8-hydroxyquinoline compounds. Zn^2+^ irreversible binding inhibitors, such as quinones, 4-dichlorocinnamic hydroxamic acid (DCHA), and 9-hydroxy-4H-pyrido [1,2-a] pyrimidin-4-one (PPO), have also been reported^18^^,^^19^. Furthermore, a long-acting Zn^2+^ active site inhibitor with slow binding kinetics has been developed^20^. However, those small molecule inhibitors are limited in clinical applications for treating intoxication of botulinum toxin due to their poor selectivity.

In the present work, the structure, binding force, and stability of RRGC tetrapeptide inhibitor was optimised by computer aided drug design, and RRGW was selected and synthesised for further investigation (Tables 2 and 3). SNAP-25 cleaved assay showed that the inhibitory activity of RRGW was higher than RRGC (Figure 2(A,B)). Rescue assay proved that RRGW partially rescued the SNAP-25 cleavage by BoNT/A LC in 3 min (Figure 2(C,D)). In this study, only the inhibitory activities of RRGW and its derivatives was compared to RRGC. Different research groups may use different toxin light chain prepared in their own lab with a large range activity, protease cleavage buffer, and readout methods, so it can produce experimental bias. DAS assay showed a significantly therapeutic effect of RRGW could not be observed on an organism level (Figure S4). Anyhow, RRGW indeed inhibited the cleavage activity of BoNT/A *in vitro* and delayed muscle paralysis *in vivo* when it was co-treated with BoNT/A.

**Figure 5.**
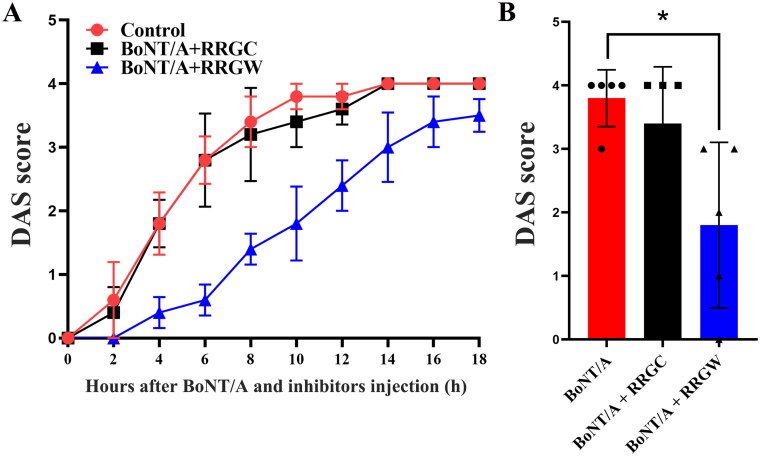
Co-injection of short peptides with BoNT/A delayed BoNT/A-induced leg muscle paralysis. (A) DAS score of the leg muscle paralysis progression within 18 h after injection with 0.75 U BoNT/A or co-injection with 0.75 U BoNT/A and 10 μL inhibitors; (B) DAS score of the leg muscle paralysis at 10 h after injection with BoNT/A or co-injection with BoNT/A and inhibitors. 500 μM RRGW and RRGC were used. There were five mice in each group, and * indicated there was significant difference between the treatment group and the control at *p* < 0.05.

Further structural optimisation and modification are needed to improve the inhibitory activity of RRGW. Mengfei Ho et al. found that glycine insertion at protease cleavage site of SNAP-25 can resist BoNT/A cleavage and promote the affinity to BoNT/A^21^. So, we mutated the C-terminal sequence of SNAP-25 (141–206) from QRAT to RRGW, RRGC and obtained two different derived peptides. Among the three mutants, QRAT is used as a random amino acid sequence control, mainly to compare and demonstrate that RRGW derived peptide has a stronger binding specificity to the toxin light chain. The results of SNAP-25 cleaved assay, inhibitor rescue assay, HPLC assay for substrates and products and DAS showed that RRGW derived peptide inhibited BoNT/A protease activity at a lower concentration by 20-fold than RRGW. It may be due to the high binding stability of RRGW derived peptide with BoNT/A LC than RRGW. To ensure the internalisation of the derived peptide into cells, a TAT transmembrane peptide was added to the N-terminal of SNAP-25 (141–206 aa) sequence. Although our data supported that a novel RRGW derived peptide was a promising inhibitor of BoNT/A, its internalisation method into the cells required further investigation. Also, those results gave cues that RRGW derived peptide might be used in combination with BoNT/A to have a different kinetic from BoNT/A in single use. The time courses of recovery from muscle paralysis with or without RRGW co-treatment will be measured in future work. RRGW delayed leg muscle paralysis when co-treatment with BoNT/A by locally used (Figure 5), and it was not preventively or systematically administrated to mice. Previously work has showed that RRGC binds to the active site of BoNT/A LC as a competitive inhibitor. In present work, RRGW was inferred that it also bound to the activity site of BoNT/A LC. Based on the result that RRGC can enter cells freely^2^, RRGW is believed that it can also enter cells by itself. However, we still could not completely exclude that RRGW derived peptide bind to the toxin prior to internalisation and be escorted into the cytosol. The short half-life of peptide drugs is a crucial factor limiting their clinical efficacy *in vivo*^22^. Compared to other peptides, the RRGW derived peptide reported in this study has more than 80 amino acids and has been shown to possess more effective inhibitory activity in DAS activity evaluations *in vivo*. In our future work, the RRGW derived peptide will be fused to Fc or HSA as long-acting carrier, or targeted delivery tags to improve its druggability.

In the present work, a novel RRGW derived peptide has been demonstrated to be a promising inhibitor of BoNT/A. Some issues such as stability, bioavailability, and systemic toxicity of RRGW derived peptide need to be investigated before it turns into a drug candidate to treat BoNT/A intoxication.

## Conclusion

In present work, a novel tetrapeptide RRGW has been found to display a higher degree of energy contribution and stability when served as a ligand binding to its receptor BoNT/A light chain. SNAP-25-cleaved assays have shown that RRGW has higher BoNT/A inhibitory activity than RRGC. In addition, co-injection of inhibitor RRGW with BoNT/A significantly delayed BoNT/A-induced muscle paralysis in the DAS assay in mice. In order to further enhance the inhibitory effect of RRGW, QRAT in the C-terminal sequence of SNAP-25 (141–206 aa) has been mutated to RRGW. SNAP-25 cleaved assay and DAS assay showed that the inhibition of RRGW derived peptide has been greatly improved than RRGC and RRGW. It will be a promising drug candidate for the treatment of BoNT/A poison.

## Supplementary Material

Supplemental Material
