# Supplementary material for: A novel RRGW derived peptide is a promising inhibitor of BoNT/A
Source: J Enzyme Inhib Med Chem. 2023 Apr 27;38(1):2203878. doi: 10.1080/14756366.2023.2203878 (PMC10150623; doi:10.1080/14756366.2023.2203878)
Supplement: Supplemental Material [file IENZ_A_2203878_SM3348.pdf]

Supporting material

Fig.S1 Cell viability of different neuroblastoma cells treated by the RRGC and RRGW, data are the average of three independent experiments.

Fig.S2 Purified BoNT/A LC and SNAP-25 were analyzed on SDS-PAGE.

Fig.S3 Statistics of the degree of posterior toe paralysis caused by different concentrations of toxins in mice. There were three mice in each group.

Fig.S4 24 hours after exposure to BoNT/A, the effects of RRGC and RRGW on leg muscle paralysis in mice. 0.75U BoNT/A and 500 μM inhibitors were used, respectively. There were five mice in each group.

Fig.S5 The HPLC and MS characteristic chromatograms of RRGC and RRGW.

Fig.S6 The HPLC and MS characteristic chromatograms of RRGL and RHGW.

Fig.S1

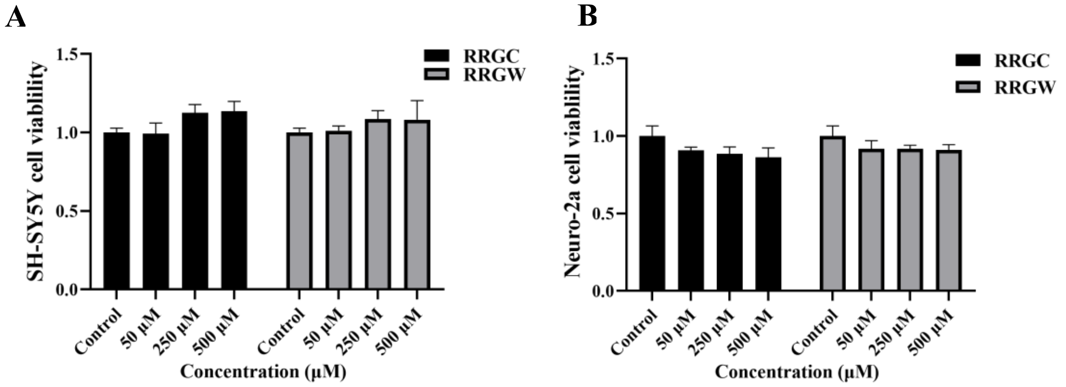
A
SH-SY5Y cell viability
1.5
1.0
0.5
0.0
RRGC
RRGW
Control
50 μM
250 μM
500 μM
Control
50 μM
250 μM
500 μM
Concentration (μM)
B
Neuro-2a cell viability
1.5
1.0
0.5
0.0
RRGC
RRGW
Control
50 μM
250 μM
500 μM
Control
50 μM
250 μM
500 μM
Concentration (μM)

Fig.S2

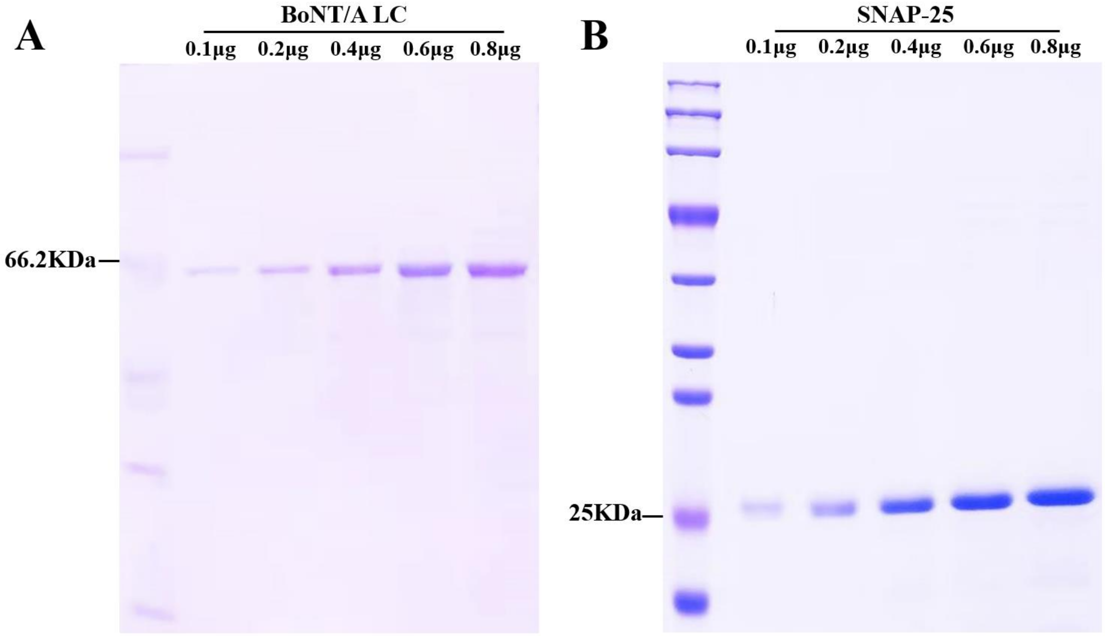
A
BoNT/A LC
0.1μg 0.2μg 0.4μg 0.6μg 0.8μg
66.2KDa
B
SNAP-25
0.1μg 0.2μg 0.4μg 0.6μg 0.8μg
25KDa

Fig.S3

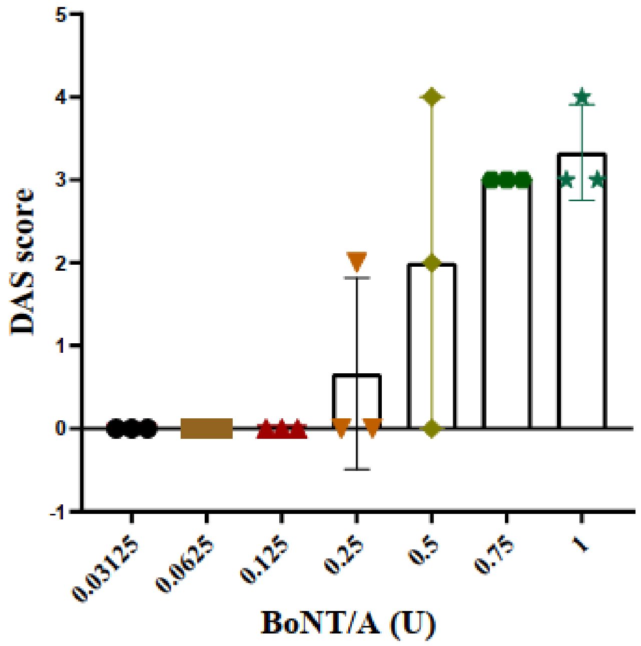

Fig.S4

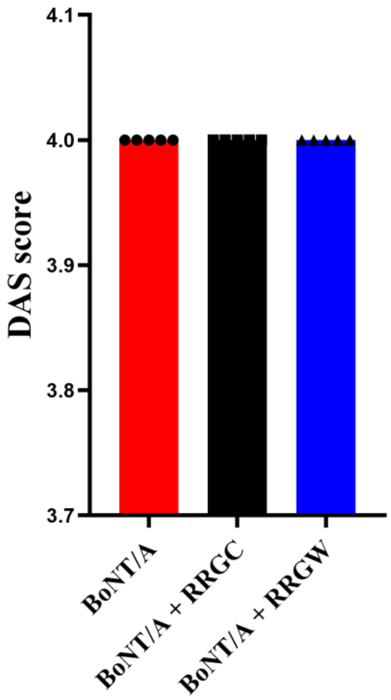

Fig.S5

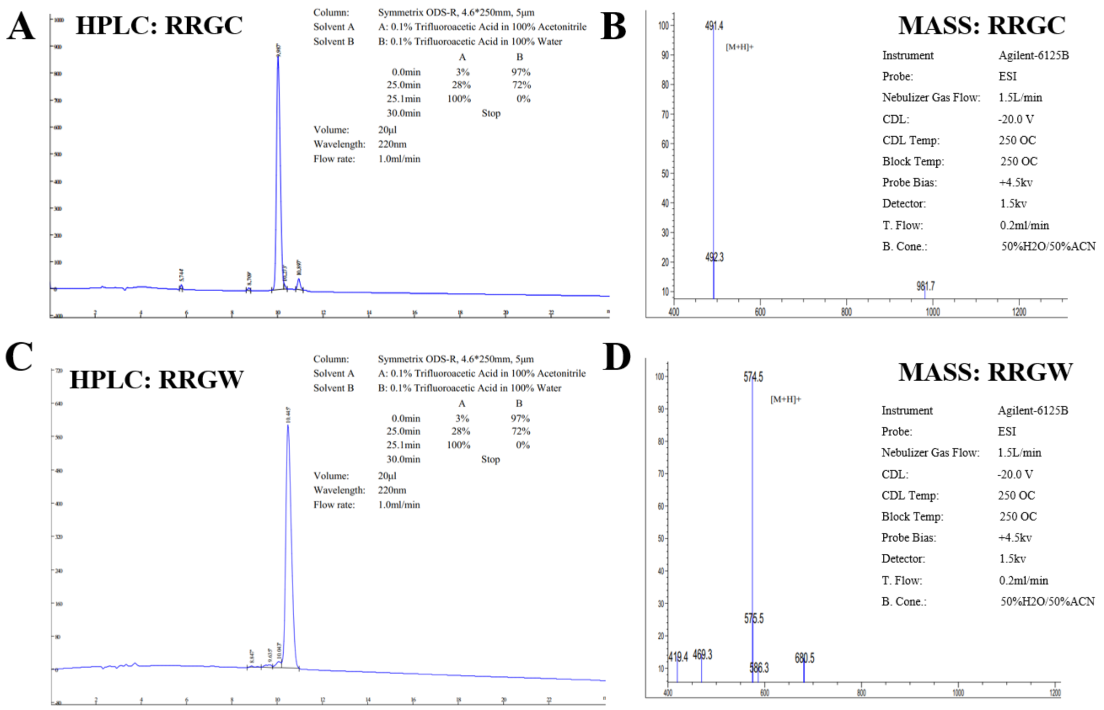
A
HPLC: RRGC
Column: Symmetrix ODS-R, 4.6*250mm, 5μm
Solvent A: A: 0.1% Trifluoroacetic Acid in 100% Acetonitrile
Solvent B: B: 0.1% Trifluoroacetic Acid in 100% Water
A B
0.0min 3% 97%
25.0min 28% 72%
25.1min 100% 0%
30.0min Stop
Volume: 20μl
Wavelength: 220nm
Flow rate: 1.0ml/min
B
MASS: RRGC
491.4
[M+H]+
492.3
981.7
Instrument Agilent-6125B
Probe: ESI
Nebulizer Gas Flow: 1.5L/min
CDL: -20.0 V
CDL Temp: 250 OC
Block Temp: 250 OC
Probe Bias: +4.5kv
Detector: 1.5kv
T. Flow: 0.2ml/min
B. Cone.: 50%H2O/50%ACN
C
HPLC: RRGW
Column: Symmetrix ODS-R, 4.6*250mm, 5μm
Solvent A: A: 0.1% Trifluoroacetic Acid in 100% Acetonitrile
Solvent B: B: 0.1% Trifluoroacetic Acid in 100% Water
A B
0.0min 3% 97%
25.0min 28% 72%
25.1min 100% 0%
30.0min Stop
Volume: 20μl
Wavelength: 220nm
Flow rate: 1.0ml/min
D
MASS: RRGW
574.5
[M+H]+
575.5
419.4
469.3
586.3
680.5
Instrument Agilent-6125B
Probe: ESI
Nebulizer Gas Flow: 1.5L/min
CDL: -20.0 V
CDL Temp: 250 OC
Block Temp: 250 OC
Probe Bias: +4.5kv
Detector: 1.5kv
T. Flow: 0.2ml/min
B. Cone.: 50%H2O/50%ACN

Fig.S6

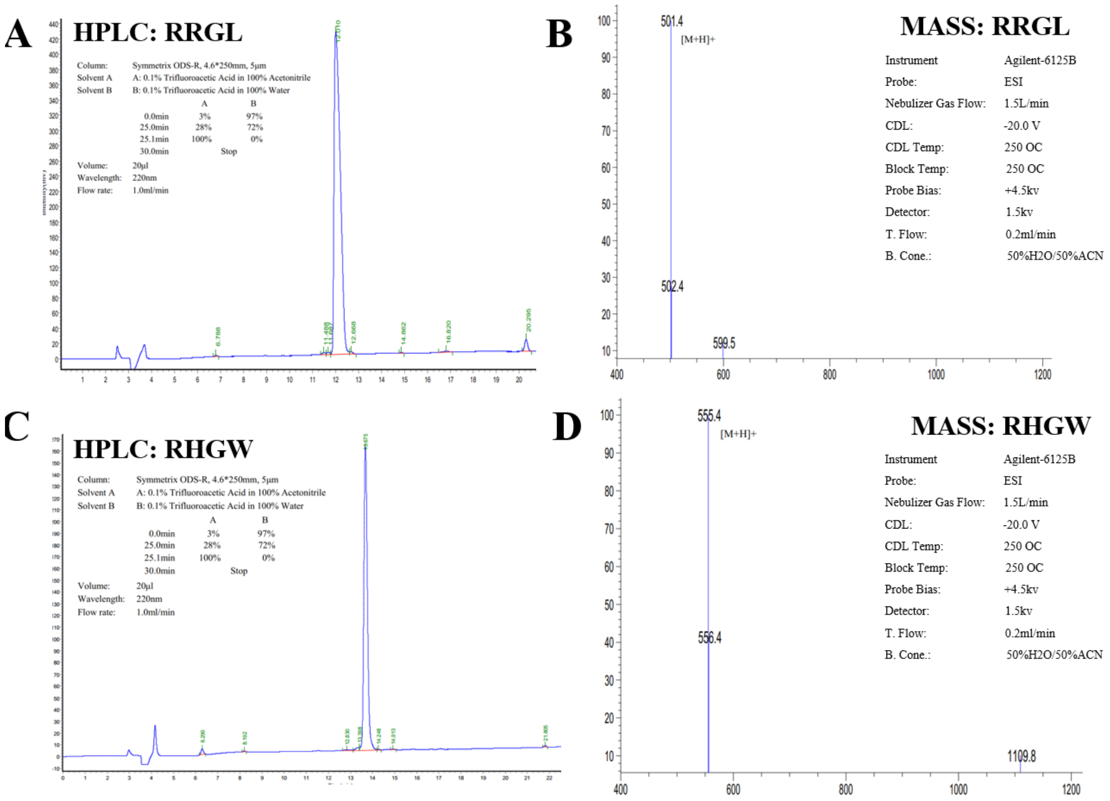
A
HPLC: RRGL
Column: Symmetrix ODS-R, 4.6*250mm, 5μm
Solvent A A: 0.1% Trifluoroacetic Acid in 100% Acetonitrile
Solvent B B: 0.1% Trifluoroacetic Acid in 100% Water
A B
0.0min 3% 97%
25.0min 28% 72%
25.1min 100% 0%
30.0min Stop
Volume: 20μl
Wavelength: 220nm
Flow rate: 1.0ml/min
B
MASS: RRGL
501.4
[M+H]+
502.4
599.5
Instrument Agilent-6125B
Probe: ESI
Nebulizer Gas Flow: 1.5L/min
CDL: -20.0 V
CDL Temp: 250 OC
Block Temp: 250 OC
Probe Bias: +4.5kv
Detector: 1.5kv
T. Flow: 0.2ml/min
B. Cone.: 50%H2O/50%ACN
C
HPLC: RHGW
Column: Symmetrix ODS-R, 4.6*250mm, 5μm
Solvent A A: 0.1% Trifluoroacetic Acid in 100% Acetonitrile
Solvent B B: 0.1% Trifluoroacetic Acid in 100% Water
A B
0.0min 3% 97%
25.0min 28% 72%
25.1min 100% 0%
30.0min Stop
Volume: 20μl
Wavelength: 220nm
Flow rate: 1.0ml/min
D
MASS: RHGW
555.4
[M+H]+
556.4
1109.8
Instrument Agilent-6125B
Probe: ESI
Nebulizer Gas Flow: 1.5L/min
CDL: -20.0 V
CDL Temp: 250 OC
Block Temp: 250 OC
Probe Bias: +4.5kv
Detector: 1.5kv
T. Flow: 0.2ml/min
B. Cone.: 50%H2O/50%ACN
